# A Review of the Dermatological Manifestations of Coronavirus Disease 2019 (COVID-19)

**DOI:** 10.1155/2020/9360476

**Published:** 2020-08-11

**Authors:** Farah Marraha, Ibtissam Al Faker, Salim Gallouj

**Affiliations:** ^1^Department of Dermatology, University Hospital Center of Tangier, Tetouan, Al Hoceima, Morocco; ^2^Faculty of Medicine and Pharmacy Tangier, Abdelmalek Essaadi University, Tangier, Morocco

## Abstract

Coronavirus disease 2019 (COVID-19) caused by severe acute respiratory syndrome coronavirus-2 (SARS-CoV-2) has affected 210 countries and territories around the world. The virus has spread rapidly, and the disease is still extending up to now. The pathophysiology for SARS-CoV-2 has not been well elucidated, and diverse hypotheses to date have been proposed. Initially, no skin manifestations were observed among patients with COVID-19, but recently a few cases have been described. In this review, we discuss these various cutaneous manifestations and skin problems related to personal protective equipment, as well as different cutaneous anti-COVID-19 drug-associated reactions. We also focus on the currently proposed managements of these rare manifestations.

## 1. Introduction

The first case infected by severe acute respiratory syndrome coronavirus-2 (SARS-CoV-2) was reported in Wuhan, China, in late November 2019. In December 2019, several patients have been presented to the hospital for acute pneumonia of unknown origin [1]. Later, SARS-CoV-2 has been found in the lower respiratory tract of hospitalized patients, and the World Health Organization (WHO) recognized the disease as COVID-19 (Coronavirus Disease 2019) on January 12, 2020. The virus has spread rapidly, and the condition is still extending up to now. On March 11, the WHO declares the COVID-19 outbreak as pandemic [2]. We are still counting to this day 11,577,979 confirmed cases with 537,103 deaths worldwide. Initially, no skin manifestations were observed among patients with COVID-19 [2, 3]. The first case was described in Thailand with petechiae rash mimicking a dengue fever [4], and then a few other cases followed [2–5]. These skin lesions can guide clinicians for diagnosis if the patients present other COVID-19 symptoms; however, viral infection cannot be the only cause; mediated inflammatory responses and drug reactions can also be suspected. The aim of our literature review is to report the various cutaneous manifestations described to date associated with COVID-19, the skin problems related to personal protective equipment, and the different cutaneous anti-COVID-19 drug reactions [6, 7]. We also discuss the different interventions proposed to manage these patients [8, 9] and solutions to protect skin and mucous membrane barriers for healthcare workers [10–12].

## 2. Epidemiology, Pathogenesis, and Diagnosis of COVID-19: An Overview

Since the epidemic began in Europe, some countries have presented virus-spreading indicators higher than others. In Italy, the first cases were detected in the last ten days of February. Its case fatality rate at present is 14.4%. In France, the mortality rate has continued to increase, reaching 17.9% (July 6, 2020). In the United States, the first cases appeared around March 10. The country represents more than 25% of the cumulative cases worldwide. As for Morocco, the first case emerged on March 2; official indicators seem to display volatile trends. It is tough to demonstrate a correlation between them. The growth factor can vary from a value of 2 to a value of 0.5 within 24 hours. The contamination rate displayed every day (new cases in 24 hours compared to the number of tests carried out in 24 hours) is also very volatile ranging from 9% on March 28 to 30% on April 8 and 2.2% on July 5. As for the mortality rate, it decreased from 6.2% to 1.6% lately (July 6, 2020) [13].

The pathophysiology of COVID-19 has not been well elucidated, but it seems similar to that of SARS-CoV [14]. SARS-CoV has the appearance of a crown under electron microscope. It is composed of genomic RNA and four structural proteins: spike (S), envelope (E), membrane (M), and nucleocapsid (N). It can attach to the host cell by binding its S protein to the receptor protein membrane [15–18]. A number of studies showed that angiotensin-converting enzyme 2 (ACE2) is a receptor on the host cell membrane, with a high affinity to SARS-CoV-2 protein (S) [19, 20]. Yet, the significance of this binding affinity is still under intensive research study [17]. ACE2 is shown to be expressed by epithelial cells of the intestine, kidney, blood vessels, and most abundantly in type II alveolar cells of the lungs [19, 20]. Importantly, Hamming et al. [20] demonstrated that ACE2 is also present in the basal cell layer of the epidermis extending to the basal cell layer of hair follicles, which might explain the cutaneous manifestation of COVID-19. Decreased ACE2 function can cause dysfunction of the renin-angiotensin system (RAS) and intensify inflammation, vascular permeability, and neutrophil accumulation [14, 21]. Rapid viral replication can also cause cellular apoptosis and triggers a cascade of inflammatory reaction, as well as an increase in cytokine and chemokine blood levels [21]. Therefore, the inflammatory response of the body also plays a crucial role in SARS-CoV-2-induced lung injury cases, since the high production of these cytokines is responsible for the accumulation of cells and fluids (cytokine storm) [18]. These cytokines arriving at the skin and reaching the various cells of the cutaneous immune system can cause the dermatological lesions described during COVID-19 infection (urticarial lesion, erythema, and vesicles) [22].

Wenzhong et al.'s latest hypothesis suggests that lung damage and inflammation are secondary to extreme lung cell poisoning [23]. In fact, ORF8, a SARS-CoV-transcribed nonstructural protein, could bind to porphyrin as well as other proteins and can attack the heme on the 1-beta chain of hemoglobin. Consequently, this will decrease hemoglobin and gas exchanges, which eventually results in ground-glass-like lung images.

Several cases of patients with COVID-19 showed leucopenia, lymphocytopenia, increased D-dimer level, and prolonged prothrombin [1, 17]. Besides, abnormal findings of different organs function assessment may indicate multiorgan failure [17]. It has been suggested that COVID-19 is a zoonotic infection as the first infected people were exposed to the wet animal market in Wuhan city [1, 17]. However, the intermediate host is still unknown. The virus is transmitted by inhalation of aerosol droplets from the infected patients [1, 17]. The fecal-oral transmission was also hypothesized [21]. Therefore, it is essential to strictly adhere to environmental and hand hygiene to control the infection [24]. So far, the golden clinical diagnosis methods to confirm cases of COVID-19 include the detection of nucleic acids in the nasal and throat swab sampling by real-time polymerase chain reaction (PCR) [25]. The identification of SARS-CoV-2-specific IgM and IgG antibodies can also be useful for diagnosis [17].

## 3. Cutaneous Manifestations in COVID-19 Patients

The most common symptoms in COVID-19 are fever, fatigue, and dry cough, succeeded by other symptoms, such as headache, nasal congestion, sore throat, myalgia, and arthralgia [1, 26]. Initially, no skin involvement during COVID infection was observed, but more recently, some cases (Table 1) have been reported [2, 4, 5, 11], as well as the skin problems related to personal protective equipment. Secondary skin reactions to the different treatments suggested are also possible.

### 3.1. Skin Lesions and COVID-19

The frequency of the skin lesions associated with COVID-19 infection varies according to the series; in a Chinese study of 1099 positive cases, the incidence was only 0.2%, while in an Italian series of 88 patients it was 20.4% [42].

Joob and Wiwanitkit described in Thailand, where the first case of COVID-19 outside of China was reported, a skin rash with petechiae in a case of COVID-19 [4]. Because of the frequent dengue cases in this country, it was the first diagnosis to be mentioned. After the appearance of other respiratory problems, COVID-19 has been diagnosed by RT-PCR. In Italy, in a series of 148 cases, Recalcati reported, after the exclusion of 60 patients who have started new drugs in 15 previous days, that 20.5% of the 88 patients developed skin manifestations [2]. Eight of the 18 (44%) had skin eruptions with symptoms in the beginning and the rest after hospitalization. Erythematous rashes were the most common sign (78%) and then urticarial (3 cases) and chickenpox-like vesicles (1 case). However, no documentation has been assembled in these cases (photos or biopsy) to prevent the spread of infection. At the end of March, the French Society of Dermatology launched a call for a case, after discovering erythematous maculopapular lesions on the faces of three patients with a very probable or confirmed COVID-19 infection [5]. Finally, 113 cases have been reported, of which 74% of them had frostbite-like lesions.

An American team with a series of 505 patients with dermatological lesions tried to study the characteristics of pernio lesion or chilblain lesions in acral skin. Despite the absence of a confirmation test of COVID-19 infection in all these patients, the results showed that 318 of them had pernio-like lesions and most of them were young. The localization of these lesions was in the feet in 84% of the cases, in the hands in 5.1% of the cases, and in both in 10% of the cases [27].

**Figure 1:**
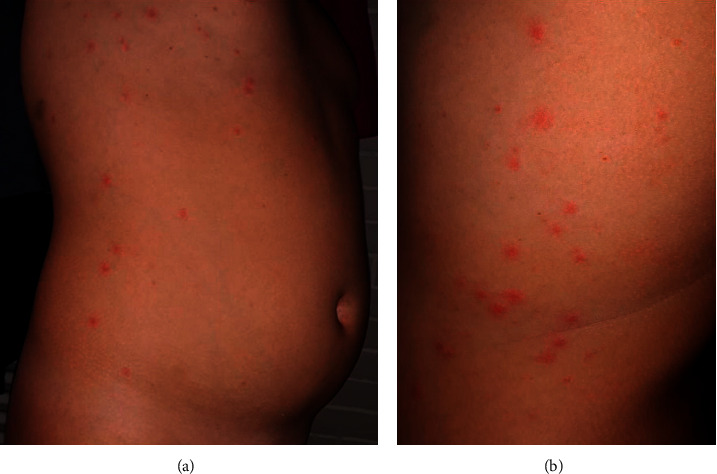
(a, b) Scattered erythematous papulovesicles on the trunk in a suspected COVID-19 patient (reprinted from Genovese et al. Varicella-like exanthem associated with COVID-19 in an 8-year-old girl: A diagnostic clue?, Pediatric Dermatology, 37(3), 435–436, Copyright (2020) [35], with permission from Wiley).

In another Spanish study [30], including 20 children and adolescents with acral lesions, four clinical patterns were described: acral erythema (30%) (Figure 1), dactylitis (20%), purpuric maculopapules (35%), and mixed pattern (15%).

RT-PCR could not confirm the presence of the virus. The authors believe that it is secondary to a realization of a diagnostic test at an early stage of the disease.

Coagulopathy and thrombocytopenia are also common complications for COVID-19 infection [17, 26, 43]. In Wuhan [26], patients with COVID-19 might present acro-ischemic lesions; there were 7 patients, in a critical situation, hospitalized in the intensive care unit with different clinical presentations, including finger/toe cyanosis, skin bulla, and dry gangrene. Four patients among them were diagnosed with definite disseminated intravascular coagulation (DIC). A team from Italy [28] tried to collect cases from a website, and 63 cases were collected, but only 54 pictures were analyzed: 31 of 54 patients had erythematous-edematous lesions and 23 of 54 had blistering lesions.

A report by Fernandez-Nieto et al. [36] describing the characterization of acute acro-ischemic lesions in 132 patients showed two different clinical presentations: chilblain-like in 72.0% of patients and the erythema multiforme-like in 28.0% of patients, all with distal localization.

**Figure 2:**
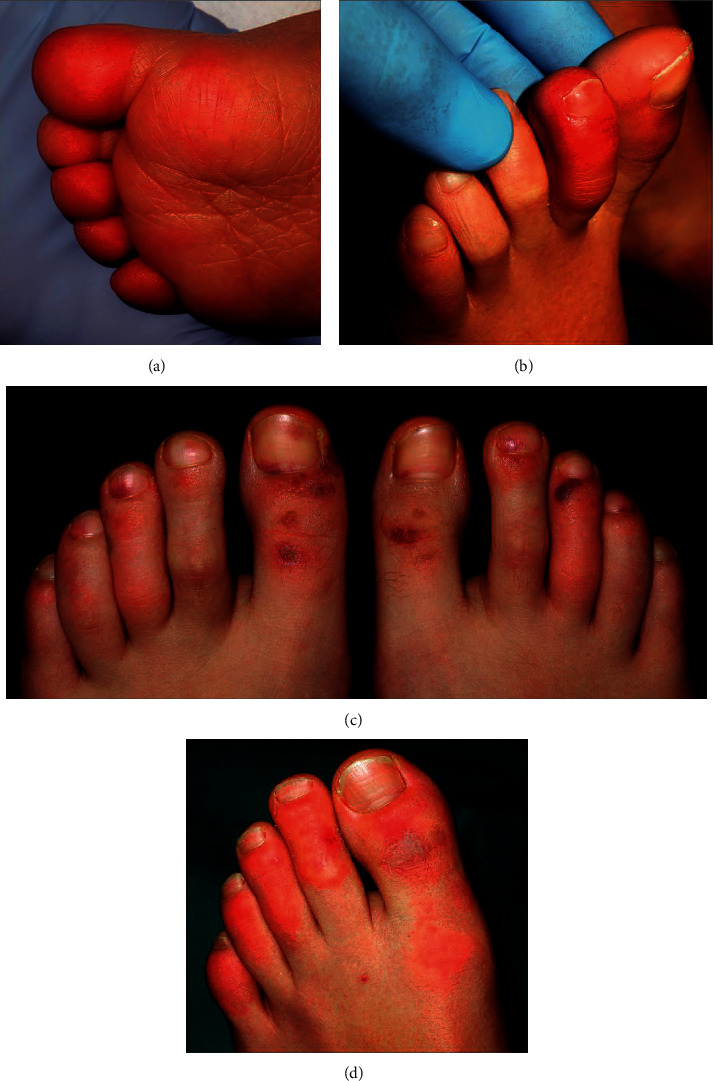
Details of the clinical spectrum (reused from Roca-Ginés et al. JAMA Dermatology 2020 [30]; reference under the terms of the CC-BY license, which permits unrestricted use, distribution, and reproduction in any medium). (a) Acral erythema pattern. (b) Dactylitis pattern. (c) Maculopapular purpuric pattern. (d) Mixed patterns.

Fernandez-Nieto et al. [31] described a patient in Madrid with an urticarial rash that appeared six days after the first symptoms. They also mentioned the presence of other cases with different skin manifestations, and for that, a prospective study is in progress. In France, Henry et al. [32] reported a similar case; however, the urticarial rash in this patient was inaugural. The only associated symptoms were odynophagia and arthralgia. This eruption was in the form of disseminated erythematous plaques with facial and acral localization. Estébanez et al. [33] published another type of cutaneous manifestation: lesions in the form of yellowish erythematous papules located in the heels. A very recent publication in Italy [34] suggested that papulovesicular eruption (varicella-like) was rare but specific to COVID-19. Twenty-two patients have been reported with the same clinical presentation; itching was present in 40.9% of patients. And the eruption was generally localized at the trunk. Another article by the same authors [35] detailed the case of a little girl who has papulovesicular skin eruption on the trunk, face, and genital tract (Figure 2); after 6 days, the patient had a moderate fever and was tested positive for SARS-CoV-2.

Sanchez et al. [37] described a new clinical presentation of skin lesions associated with COVID-19. They reported a skin eruption similar to that of pityriasis rosea: erythematous and squamous plaque, in the trunk, upper arms, and periumbilical.

A Spanish publication has classified the skin lesions observed during COVID-19 into 5 types: maculopapular eruptions in 47% of cases, urticarial lesions in 19% of cases, acral areas of erythema with vesicles or pustules in 19%, other vesicular eruptions in 9%, and livedo or necrosis in 6% [29].

In the pediatric population, the frequency of coronavirus infection is estimated to be less frequent than adults (<1%) [22, 44]. A particular presentation has been reported in children in Europe and North Africa, with a lesion similar to Kawasaki syndrome [38, 44, 45].

Hence, the WHO established a preliminary definition and case for multisystem inflammatory disorder in children and adolescents [44] to help define and recognize these cases for better management and possible surveillance.

Clinical presentation includes fever, rash, mucosal inflammation associated with gastrointestinal problems, myocardial dysfunction, and sometimes even hypotension or shock [44].

According to a French series, concerning 16 children with this syndrome, the skin rash was present in 81% of patients, edema with plantar and palmar redness was present in 67% of patients, and 87% of them had dry lips [38].

As part of this syndrome, the skin eruption can be very varied; for example, an erythema multiforme rash has been described in a 13-year-old patient [39].

Other lesions have also been reported, such as androgenic alopecia (AAG) [40], sebopsoriasis, herpes, and exanthem [41]. Also, nonspecific erythematous lesions in the trunk and face have been described [2].

### 3.2. Skin Damages among Healthcare Workers

To control this infection, all personnel must wear personal protective equipment (PPE) for an extended period in addition to other safety and health measures. Eczema is the most common problem among healthcare workers [10], often secondary to frequent hand hygiene and long-time gloves wearing [46]. Besides, humidity, prolonged contact with masks, and goggles may cause a variety of cutaneous diseases such as contact and pressure urticaria or contact dermatitis and pigmentation of the nasal bridge [3, 11]. In a survey study [12], Lan et al. found that 526 participants among healthcare professionals mentioned that they had damaged skin. The nasal bridge was the most affected area and then hands, cheek, and forehead. Dryness and desquamation were the most common symptoms, and these damages depended on the hours of work, especially for gloves wearing.

**Figure 3:**
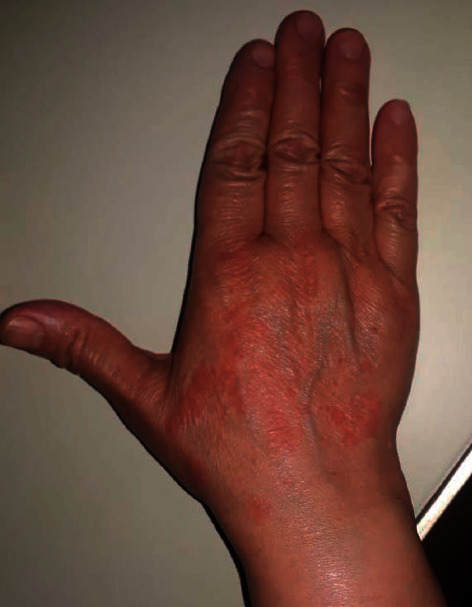
Contact dermatitis caused by chemical materials in latex gloves (picture is from our department; written consent was provided by the participant).

Another questionnaire distributed among 66 patients objected that wearing of masks has caused in 95.1% of cases side effects including first, nasal bridge scar. Wearing of gloves has caused in 88.5% of cases skin reactions [47]. We are currently carrying out a study to clarify the consequences of wearing personal protective equipment on the skin. To date, 268 health personnel have completed the questionnaire. Because of frequent handwashing to prevent the disease, dry skin was present in 68.3%, with erythema in 31.3% of cases (Figure 3).

### 3.3. Aggravation of Previous Skin Diseases during COVID-19

Exacerbations of preexisting dermatoses such as acne, rosacea, atopic dermatitis, and neurodermatitis have been observed [3, 7]. This can be explained by the prolonged wearing of masks during the epidemic. The occlusion generated by protective hats may provoke pruritus and folliculitis or exacerbate seborrheic dermatitis [11], and the frequent use of disinfectant and soap can impair the hydrolipid mantle of the skin and increase the risk of contact dermatitis [7]. The current stress generated by the global situation and confinement can also exacerbate dermatoses [12].

### 3.4. Skin Reactions Associated with Different COVID-19 Treatments

Preventive measures are currently the best strategy to fight COVID-19. While vaccines and monoclonal antibodies against SARS-CoV-2 are under development, several other therapies are proposed [48, 49], hence the interest to review the skin side effects of these molecules. Chloroquine/hydroxychloroquine (HCQ), a widely used antimalarial and autoimmune disease drug, has been demonstrated to inhibit SARS-CoV-2 and blocks viral activity by increasing endosomal pH [48–50]. However, the use of this drug remains controversial; some studies have tried to prove the effectiveness of this treatment [48, 50–52]. Nevertheless, each had methodological deficiencies [53]. Other studies are underway with a large sample. Chloroquine is widely used in dermatology disorders and autoimmune disease; its toxicity is infrequent [54], and reactions can be mainly gastrointestinal and cutaneous (pruritus or urticaria), usually mild [54, 55]. Few cases of HCQ toxidermia have been reported. Acute generalized exanthematous pustulosis (AGEP) was the most common presentation [56, 57]; it appears like a sterile nonfollicular pustule with an erythematous base [58]. Also, drug reactions with eosinophilia and systemic symptoms (DRESS) have been described in four reports [58]. Besides, rare cases of HCQ and Sweet syndrome have also been reported [6, 59]. The Moroccan Health Ministry and National Technical Committee for Prevention and Control of COVID-19 decided on March 23, 2020, to adopt the therapeutic protocol based on using hydroxychloroquine under medical supervision, and no problem has been noticed yet. Other prescription drugs such as OTC, antibiotics, healthcare products, and a variety of plants can also cause a skin reaction. Zheng and Lai [7] reported cases of urticaria, urticarial vasculitis, and other pruritus lesions in coronavirus pneumonia patients after accepting anti-COVID-19 medicines.

## 4. Management of Dermatological Manifestations Associated with COVID-19

Healthcare institutions and policymakers developed various plans to control the pandemic, including preparing hospitals and clinics, developing a strategy for the identification of suspected COVID-19 cases, and implementing a strategy to reduce the spread [24]. Prevention of nosocomial infection should be another priority; this will require specialized protocols and training of the medical staff. In most countries, several dermatological services have been transformed into a structure for the treatment and isolation of patients with COVID-19 infection, and many private dermatology practices have closed doors [3]. Different solutions have been proposed.

### 4.1. Patient Management

During the COVID-19 epidemic in China, Wang et al. [60] reported a series of 138 hospitalized patients (41.3%) were presumed to have been infected at the hospital, and 29% of them were healthcare workers. For new patients and outpatients, a preexamination and triage stations at the hospital entrance have been proposed to minimize nosocomial infection and contact [3, 61–63]. Digital technology seemed like a right solution for nonemergency patients [3, 61, 62]. A recently published Swiss study before the COVID-19 outbreak [64] revealed that over 75% of the patients were interested in using telehealthcare (medical consultations and electronically transmitted prescriptions). During a patient visit, the dermatologist and the patient should both wear a surgical mask with hand hygiene. The dermoscopy should be performed with caution and only if necessary to prevent dermatoscope from becoming a possible source of nosocomial spread [65]. In addition, this exam should be avoided for specific sites such as hands, nails, face, eyes, and mucosa, and for all patients with signs and symptoms suggestive of COVID-19 [65].

Several skin diseases, such as psoriasis, as well as depression and anxiety, can increase the risk of 2019-nCoV infection [61, 66]. Phototherapy and the other hospital treatments are needed to be adjusted to avoid displacement [7]. Up until today, there is not enough evidence or guidelines for managing the patients with severe inflammatory skin disorders treated with immunomodulators during this pandemic, and there is no proof that these persons may have a more increased risk of becoming infected with COVID-19 [8, 9, 61]. The first treatment option in this particular period must be the one who has lower effects on personal immune functions such as IL-17 inhibitor [7]. Other treatment options include decreasing the dosage of traditional immunosuppressants (methotrexate and cyclosporine) [8]. On the other hand, there has not been any recommendation or indication justified to stop or reduce the systemic corticosteroid dose [8].

In the case of skin cancer, surgical treatment, if indicated, should not be delayed, especially in critical localization [67].

### 4.2. Preventive Measures for Cutaneous Complications Related to Personal Protective Equipment

The wearing of the PPE for an extended period and the cutaneous complications that generate with stress and anxiety can reduce the efficiency of the health workforce [10]. As such, it has been recommended to use latex-free gloves or use of cotton gloves inside it to avoid cutaneous hand problems. Moreover, the use of cleansing products containing moisturizing ingredients and the application of hand cream frequently are highly recommended [3, 11, 68]. The alcohol hand sanitizers may be used more frequently as they have been shown to be less irritating than washing hands with soap [69]. To avoid friction and pressure from wearing the mask, it has been suggested to wear a properly fitted mask, in different ways, and apply moisturizers or gel just before [10, 11], or combine a paper towel with facial mask [67].

### 4.3. Medical Education and Care in Dermatology

The medical training of future doctors and the scientific communication between dermatologists have also been affected. In Morocco, the Ministry of National Education has deployed an online platform for continued remote instruction for students. The Department of Dermatology and Allergology of the Ludwig Maximilian University Hospital [70] has presented numerous online case studies and offered opportunities for continuous education. Other similar projects with different features and focus already exist on the Internet [71].

## 5. Conclusion

To date, several skin manifestations related to COVID-19 have been reported, but additional efforts are needed to collect further data. It is essential not to forget the patients who suffer from other skin diseases and to insist on preventive measures for healthcare professionals.

## Figures and Tables

**Table 1:** Summary of the published dermatological findings in COVID-19 patients.

References	Journal	Country of origin	Number of cases	Dermatological findings
Joob and Wiwanitkit, 2020 [4]	*Journal of the American Academy of Dermatology*	Thailand	1	Petechiae rash
Recalcati et al., 2020 [2]	*Journal of the European Academy of Dermatology and Venereology*	Italy	18	Erythematous rash, widespread urticaria, and chickenpox-like vesicles
Zhang et al., 2020 [26]	*Zhonghua Xue Ye Xue Za Zhi (in Chinese)*	China	7	Acro-ischemia
Beylot-Barry, 2020 [5]	*French Society of Dermatology website*	France	113	Erythematous maculopapular lesions on the face and pseudofrostbite
Freeman et al., 2020 [27]	*Journal of the American Academy of Dermatology*	USA	318	Pernio-like lesions
Piccolo et al., 2020 [28]	*Journal of the European Academy of Dermatology and Venereology*	Italy	54	31 of 54 had erythematous-edematous
				23 of 54 had blistering lesions
Galván Casas, 2020 [29]	*British Journal of Dermatology*	Spain	375	Maculopapular eruptions
				Urticarial lesions
				Acral areas of erythema with vesicles or pustules
				Other vesicular eruptions
				Livedo or necrosis
Roca-Ginés et al., 2020 [30]	JAMA Dermatology	Spain	20	Acral erythema
				Dactylitis
				Purpuric maculopapules
				Mixed pattern
Fernandez-Nieto et al., 2020 [31]	*Journal of the European Academy of Dermatology and Venereology*	Spain	1	Urticarial rash
Henry et al., 2020 [32]	*Journal of the European Academy of Dermatology and Venereology*	France	1	Inaugural urticarial rash
Estébanez et al., 2020 [33]	*Journal of the European Academy of Dermatology and Venereology*	Spain	1	Erythematous-yellowish papules
Marzano et al., 2020 [34]	*Journal of the American Academy of Dermatology*	Italy	22	Varicella-like papulovesicular exanthem
Genovese et al., 2020 [35]	*Pediatric Dermatology*	Italy	8	Papulovesicular skin eruption
Fernandez-Nieto et al., 2020 [36]	*Journal of the American Academy of Dermatology*	Spain	95	Chilblain-like lesions
			37	Rounded erythematous macules and vesicles
Sanchez et al., 2020 [37]	*JAMA Dermatology*	France	1	Squamous and erythematous patch
Pouletty et al., 2020 [38]	*Annals of the Rheumatic Diseases*	France	16	Skin rash (Kawasaki-like syndrome), edema with plantar and palmar redness
Bapst et al., 2020 [39]	*BMJ Case Reports*	Switzerland	1	Erythema multiforme
Goren et al., 2020 [40]	*Journal of Cosmetic Dermatology*	USA	41	AGA
Gaspari et al., 2020 [41]	*Journal of the European Academy of Dermatology and Venereology*	Italy	20	Sebopsoriasis
				Facial herpes
				Exanthem
				Acral vasculitic eruption

## Data Availability

No data were used to support this study.
